# Antibiotic use prior to a lung cancer diagnosis: a population-based study

**DOI:** 10.1007/s10552-021-01413-5

**Published:** 2021-03-22

**Authors:** Lukas Löfling, Shahram Bahmanyar, Helle Kieler, Mats Lambe, Gunnar Wagenius

**Affiliations:** 1 Centre for Pharmacoepidemiology, Division of Clinical Epidemiology, Department of Medicine – Solna, Karolinska Institutet, Solna, Sweden; 2 Etiological Research Unit, Department of Research, Cancer Registry of Norway, Oslo, Norway; 3 Clinical Pharmacology, Department of Laboratory Medicine, Karolinska Institutet, Huddinge, Sweden; 4 Regional Cancer Centre Central Sweden, Uppsala, Sweden; 5 Department of Medical Epidemiology and Biostatistics, Karolinska Institutet, Solna, Sweden; 6 Cancer Theme, Karolinska University Hospital, Solna, Sweden

**Keywords:** Lung cancer, Antibiotics, Early symptoms, Sweden, Pneumonia

## Abstract

**Aim:**

To examine patterns of recent pre-diagnostic fillings of antibiotics as an indicator of early symptoms of lung cancer.

**Methods:**

Individuals diagnosed with lung cancer (cases) in 2009–2016 were identified in the Swedish National Lung Cancer Register, a population-based register, and randomly matched with up to five individuals free of lung cancer (controls) from the general population. Conditional logistic models were used to estimate odds ratios for the association between lung cancer and a recent history of filled antibiotic prescriptions.

**Results:**

The study included 27,017 cases and 129,355 controls. The likelihood of recent exposure was approximately two times higher in cases compared to controls. The magnitude of the effect size became more pronounced with proximity to the diagnosis of lung cancer and an increasing number of filled prescriptions. While the magnitude of the effect size did not differ by sex or educational level, it became attenuated with increasing age. There was no evidence supporting a trend in the magnitude of the effect size for the association between lung cancer and a history of repeated fillings by cancer stage.

**Conclusion:**

Lung cancer was associated with an increased likelihood of a recent history of filled antibiotic prescriptions. However, there was no evidence of an association between repeated fillings and a diagnostic delay, as reflected by stage. Our findings underscore the importance of clinical reassessment to rule out lung cancer following pneumonia treatment, especially for patients with multiple treatment cycles.

**Supplementary Information:**

The online version contains supplementary material available at 10.1007/s10552-021-01413-5.

## Introduction

At present, screening for lung cancer is only recommended in three high-income countries (United States of America, Canada, and Japan), focusing mainly on high-risk groups such as long-term smokers [1]. Today, approximately 50% of patients with lung cancer in Western countries are diagnosed with late-stage cancer [2]. Because pneumonia can be an early symptom and is the most common differential diagnosis [3, 4], it is of interest to examine patterns of antibiotic use prior to the diagnosis of lung cancer.

The few studies to date that have examined histories of antibiotic use in patients with lung cancer have mainly focused on possible causal associations, i.e., antibiotics as a risk factor or risk modifier [5, 6]. In these studies, information on antibiotic use in the period directly before the date of lung cancer diagnosis has generally been excluded to avoid the influence of reversed causality.

Using routinely collected data, we aimed to compare patterns of recent fillings of prescriptions of antibiotics (i.e., within three years prior to the diagnosis of lung cancer) recommended for the treatment of pneumonia between patients with lung cancer and control individuals free of lung cancer. We also explored possible trends in the magnitude of the effect size for the association between a history of repeated pre-diagnostic fillings of antibiotic prescriptions and lung cancer by stage as a possible indicator of diagnostic delays.

## Materials and methods

### Study design and study population

We performed a population-based study with routinely collected data to examine possible associations between a diagnosis of lung cancer and a recent history of filled prescriptions of antibiotics recommended for the treatment of pneumonia (Supplementary Table S1). We identified individuals diagnosed with lung cancer (International Classification of Diseases for Oncology third edition [ICD–O–3] code: C34 or ICD–7 code: 1,621) between 2009 and 2016 (cases), as recorded in the Swedish National Lung Cancer Register (NLCR). The NLCR includes 97% of all individuals registered with a diagnosis of lung cancer in the Swedish Cancer Register (SCR) to which reporting is mandated [7, 8]. Using a risk set sampling approach, each case was individually matched with up to five randomly selected individuals free of lung cancer (controls), at the date of the diagnosis for the case, from the Swedish Population Register [9]. The matching factors were the year of birth, sex, and place of residence at the date of diagnosis for the case. The index date was defined as the date of the first lung cancer diagnosis recorded in the NLCR or the SCR, and the corresponding date for the matched controls.

We excluded cases with unclassified, unknown, or missing lung cancer histopathology, cases with more than a six-month difference between the recorded date of diagnosis in the NLCR and the SCR, and controls with a record of a lung cancer diagnosis in the SCR before the index date.

### Data sources

Data for the present study were retrieved from the Lung Cancer Database Sweden (LCBaSe) [2, 10], a register-based research database generated by record linkages between the NLCR, the SCR, the National Patient Register (NPR), the Prescribed Drug Register (PDR), the Cause of Death Register (CDR), the Multi-generation Register (MGR), the Swedish Population Register, and the LISA-database (a database containing sociodemographic data). The record linkages were made possible by the use of the personal identity number, a unique personal identifier given to all residents of Sweden [9]. In this study, we used data in the LCBaSe from the NLCR, the SCR, the NPR, the PDR, the Swedish Population Register, and the LISA-database [8, 9, 11–14].

### Definition of exposure

We used four different measures to assess the history of filled prescriptions of antibiotics recommended for treatment of pneumonia (Supplementary Table S1) as recorded in the PDR within three years prior to the index date:

1. at least one filled prescription,
2. repeated fillings (≥ 2 fillings),
3. the number of filled prescriptions (categorized as 0, 1, 2, 3, and ≥ 4), and
4. the number of different types of antibiotics (unique anatomical therapeutic chemical codes at the 5th level, categorized as 0, 1, 2, and ≥ 3).

### Statistical analysis

Descriptive analyses were used to describe the clinical characteristics and demographics of cases and controls. Continuous variables were summarized using the median and the first and third quartiles (q1 and q3). Categorical variables were summarized with counts and per cent.

We used conditional logistic regression models, with the matched individuals as the clusters, to estimate odds ratios (OR) and 95% confidence intervals (CI) for possible associations between a diagnosis of lung cancer (non-small cell lung cancer [NSCLC] overall, squamous cell carcinoma [SCC], adenocarcinoma, and small cell lung cancer [SCLC]) and a history of recent exposure. The effect of being a patient with lung cancer was estimated for the different histopathological subtypes overall and, within each histology, separately by sex, age, educational level, summarized cancer stage according to the tumor–node–metastasis [TNM] system from the American Joint Committee on Cancer [15], and separately for the descriptors that make up the stage. In the adjusted model, the highest attained educational level the year before the index date (categorized by years of formal education: ≤ 9, 10–12, and ≥ 13; baseline level: “ ≤ 9”) was used as an indicator of socioeconomic status. Other covariates included in the adjusted models were a record in the NPR of a chronic obstructive pulmonary disease (COPD) diagnosis (ICD–10: J44, baseline level: “no”) in the five years prior to the index date, at least one filled prescription of pre-defined antibiotics at any time prior to the start of the observation period (a minimum of six months) (baseline level: “no”), a history of any cancer (any record of a diagnosis in the SCR prior to the index date) (baseline level: “no”), and the other TNM-descriptors in the descriptor specific subanalyses (e.g., if estimating the effect of exposure in subgroups of the T-descriptor then the estimate was adjusted for the N- and M-descriptors; baseline levels: “T1” and “N1”).

We performed several sets of sensitivity analyses. First for different time intervals of the observation period, and second for those with an index date of the 1st July 2009 or after and without records of filled prescriptions of a pre-defined antibiotic within 12 months prior to the start of the observation period (washout period of 12 months) (Supplementary Figure S1). Third, where filled prescriptions in the three months before the index date were not considered, and fourth, following the exclusion of individuals with a history of COPD.

All data management and statistical analyses were performed using R statistical packages version 3.5.3 (R Development Core Team) and Stata version 15 (StataCorp LP), or later versions.

## Results

We identified a total of 29,890 patients with a diagnosis of lung cancer between 2009 and 2016 (Supplementary Figure S2). Of these, 2,873 cases (and their matched controls) were excluded because of unclassified, unknown or missing histopathology (*n* = 2275) or more than a six-month difference between the recorded date of diagnosis in the NLCR and the SCR (*n* = 598). An additional 90 controls were excluded because of a record of diagnosis of lung cancer in the SCR before their index date. Hence, the final study population encompassed a total of 27,017 patients with lung cancer and 12,9355 controls.

### Characteristics of the study population

A high educational level (i.e., post-upper secondary degree) was less common among cases (16%) compared to controls (25%) (Table 1). Compared to cases, a lower proportion of controls had a history of COPD (13% and 3%, respectively).

**Table 1:** Baseline characteristics of patients with a diagnosis of lung cancer (cases) and individuals free of lung cancer (controls), Lung Cancer DataBase Sweden, 2009–2016

Sex		Lung cancer status			
		Lung cancer		Free of lung cancer (controls)	
		(*n* = 27,017)		(*n* = 129,355)	
	Men	13,477	(49.9)	64,171	(49.6)
	Women	13,540	(50.1)	65,184	(50.4)
Age at diagnosis in cases and the corresponding age for matched controls (years)	Median (q1,q3)	70.0	(64.0, 76.0)	70.0	(64.0, 76.0)
	< 50	620	(2.3)	3085	(2.4)
	50–59	2670	(9.9)	13,198	(10.2)
	60–69	9381	(34.7)	45,353	(35.1)
	70–79	10,199	(37.8)	48,162	(37.2)
	≥ 80	4147	(15.3)	19,557	(15.1)
Educational level^a^					
	Low	10,907	(40.4)	42,996	(33.2)
	Middle	11,371	(42.1)	52,610	(40.7)
	High	4350	(16.1)	32,199	(24.9)
	Missing	389	(1.4)	1550	(1.2)
Stage at diagnosis^b^					
	I–II	5922	(21.9)	NA	NA
	III	5842	(21.6)	NA	NA
	IV	14,948	(55.3)	NA	NA
	Missing	305	(1.1)	NA	NA
Histopathology					
	SCLC	3868	(14.3)	NA	NA
	NSCLC	23,149	(85.7)	NA	NA
Comorbid lung conditions^c^					
	COPD	3383	(12.5)	3463	(2.7)
	Asthma	641	(2.4)	2861	(2.2)
Number of filled prescriptions of antibiotics recommended for pneumonia^d^					
	0	13,533	(50.1)	86,787	(67.1)
	1	6732	(24.9)	25,633	(19.8)
	2	3290	(12.2)	9374	(7.2)
	3	1621	(6.0)	3761	(2.9)
	≥ 4	1841	(6.8)	3800	(2.9)

Cases and controls were matched by sex, year of birth and place of residence

*q1* First quartile, *q3* Third quartile, *COPD* Chronic obstructive pulmonary disease, *SCLC* Small cell lung cancer, *NSCLC* Non-small cell lung cancer, *NA* Not applicable

Data are median (q1, q3) or n (%)

^a^Highest attained educational level the year before the index date (i.e., date of lung cancer diagnosis and the corresponding date for the matched individuals free of lung cancer), categorized by years of formal education: ≤ 9 (low, mandatory), 10–12 (middle, upper secondary), and ≥ 13 (high, post-upper secondary)

^b^Based on the tumor–node–metastasis (TNM) classification system

^c^Within five years prior to the index date (i.e., date of lung cancer diagnosis and the corresponding date for the matched individuals free of lung cancer), based on data from the National Patient Register

^d^Based on filled prescriptions of antibiotics recommended for the treatment of pneumonia as recorded in the Prescribed Drug Register within three years before the index date, i.e., date of lung cancer diagnosis and the corresponding date for the matched individuals free of lung cancer

### Patterns and odds of fillings

**Fig. 1:**
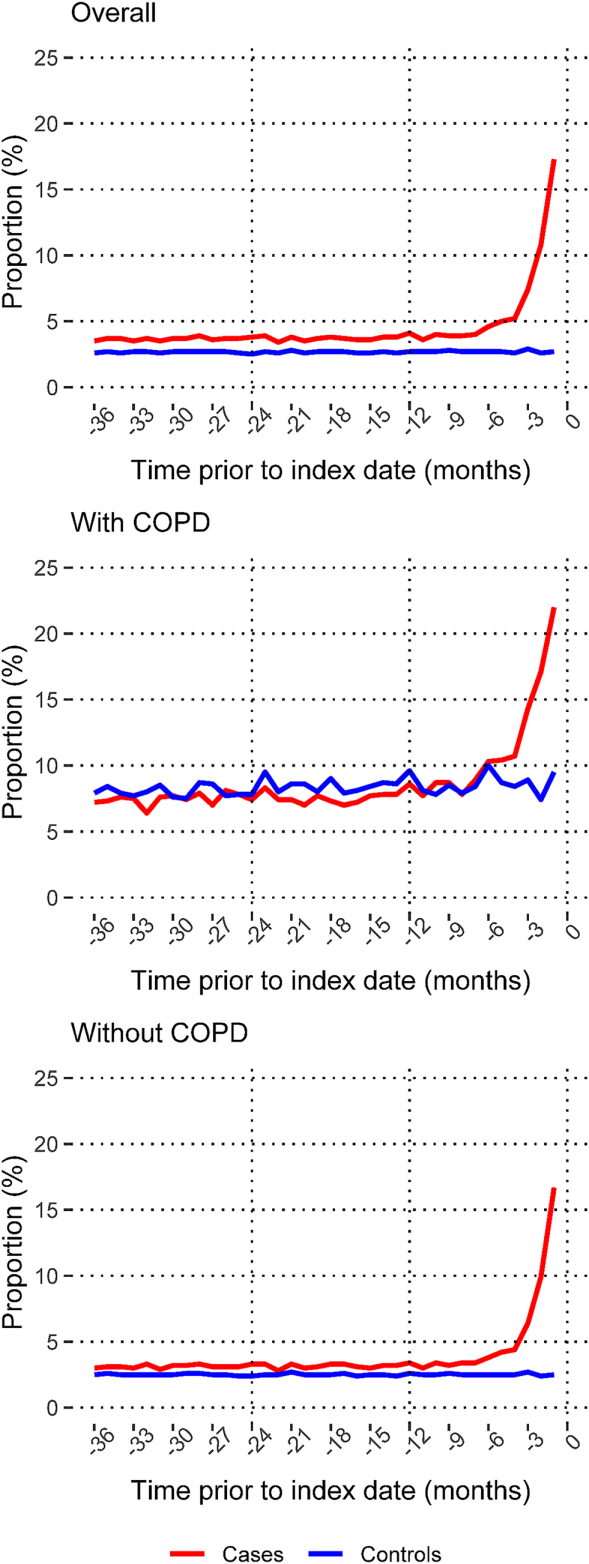
The proportion of cases (individuals with lung cancer) and controls (individuals free of lung cancer) with at least one filled prescription of antibiotics recommended for the treatment of pneumonia. Presented for separate months of the observation period, overall and by history of chronic obstructive pulmonary disease. Cases and controls were matched by sex, year of birth and place of residence. Lung Cancer DataBase Sweden, 2009–2016

Half of the cases and one-third of the controls had a recent history of at least one filled prescription of antibiotics (Table 1). The proportion of cases and controls exposed to antibiotics did not vary substantially by age. For cases, the proportion varied between 47% (≥ 80 years) and 52% (60–69 years), and for controls, between 30% (< 50 years) and 34% (60–69 years). Among patients with lung cancer, the percentage with at least one filled prescription started to increase around three to four months prior to the diagnosis, while it remained relatively stable for individuals free of lung cancer, this was independent of a history of COPD (Fig. 1). In patients with lung cancer, ever-smokers had more often filled at least one prescription compared to never-smokers, an observation that was present throughout the observation period (Supplementary Figure S3). The proportion of individuals with repeated fillings was 25% among patients with lung cancer and 13% among individuals free of lung cancer (Table 1), and the maximum treatment cycles were 63 and 78, respectively. In a comparison between individuals with and without a history of COPD, both cases and controls with COPD had more often filled repeated prescriptions of antibiotics (Supplementary Figure S4).

**Fig. 2:**
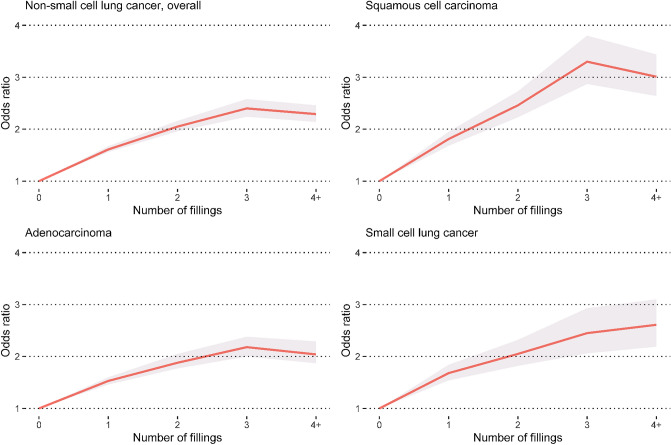
Odds ratios (solid line) and 95% confidence intervals (shaded area) for the association between a diagnosis of lung cancer and the number of recently filled prescriptions of antibiotics recommended for the treatment of pneumonia. Cases and controls were matched by sex, year of birth and place of residence. The odds ratios were adjusted for sex, year of birth, place of residence, highest attained education, previous chronic obstructive pulmonary disease diagnosis, previous use of antibiotics recommended for the treatment of pneumonia, and history of any cancer. Lung Cancer DataBase Sweden, 2009–2016

**Fig. 3:**
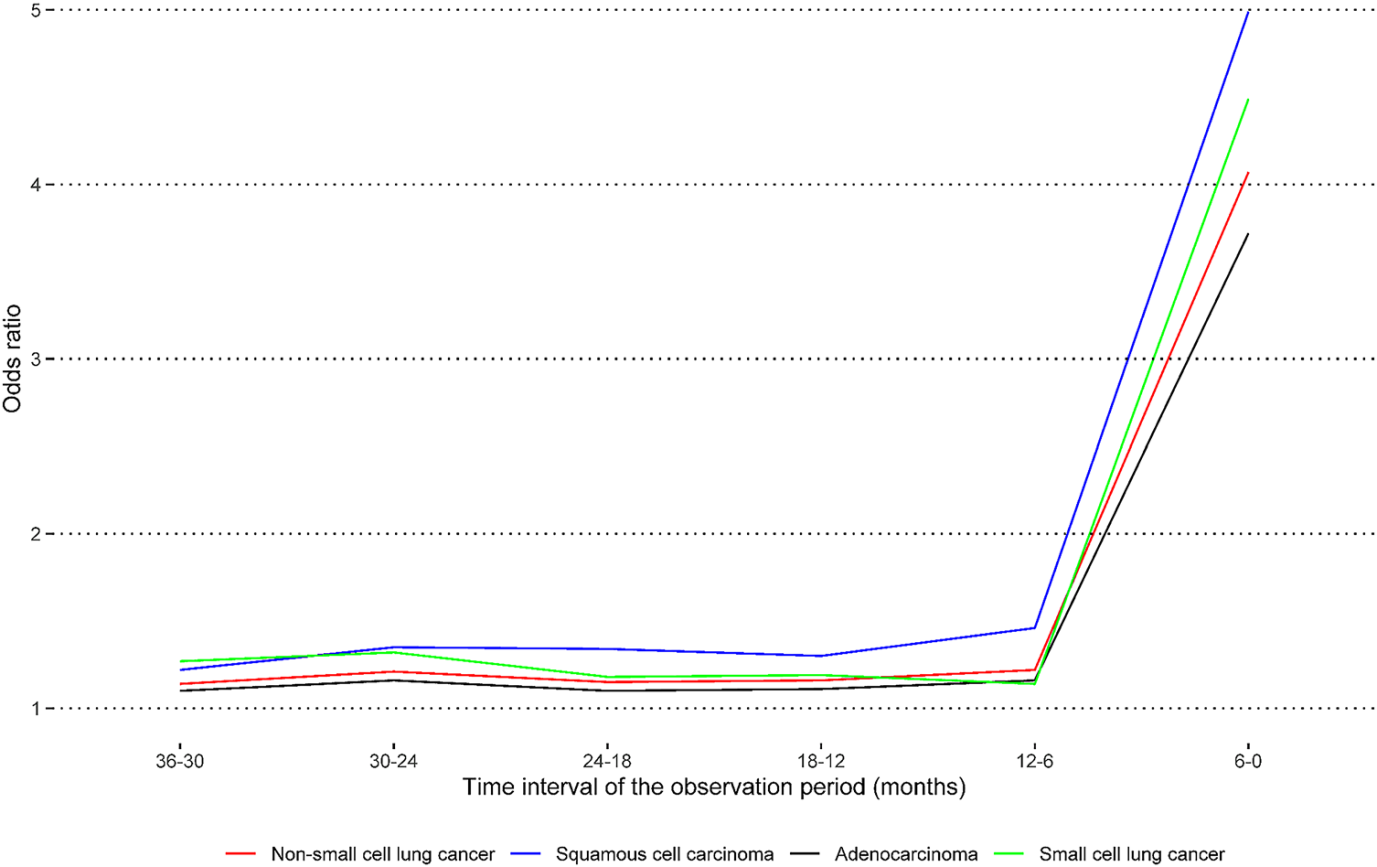
Odds ratios for the association between a diagnosis of lung cancer and a recent history of at least one filled prescription of antibiotics recommended for the treatment of pneumonia in different time intervals before diagnosis. Cases and controls were matched by sex, year of birth and place of residence. The odds ratios were adjusted for sex, year of birth, place of residence, highest attained education, previous chronic obstructive pulmonary disease diagnosis, previous use of antibiotics recommended for the treatment of pneumonia, and history of any cancer. Lung Cancer DataBase Sweden, 2009–2016

Compared to controls, the likelihood of a recent history of at least one filled prescription was elevated in patients with lung cancer: NSCLC overall (OR: 1.83, 95% CI 1.77–1.88), SCC (OR: 2.18, 95% CI 2.05–2.32), adenocarcinoma (OR: 1.70, 95% CI 1.64–1.77), and SCLC (OR: 1.92, 95% CI 1.78–2.06) (Table 2). The magnitudes of these effect sizes became more pronounced with an increasing number of filled prescriptions (Fig. 2) and with proximity to the diagnosis of lung cancer (Fig. 3, Supplementary Table S2). While the magnitudes of the effect sizes did not differ by sex or educational level, they became attenuated with increasing age (Table 2). Compared to controls, patients with lung cancer had between two and three times higher odds for a recent history of four or more filled prescriptions in three years prior to the diagnosis. A similar pattern was observed for the association with the number of different types of antibiotics from filled prescriptions (Supplementary Table S3).

**Table 2:** Odds ratios and 95% confidence intervals for the association between a diagnosis of lung cancer and a recent history of at least one filled prescription of antibiotics recommended for the treatment of pneumonia, Lung Cancer DataBase Sweden, 2009–2016

NSCLC, Overall	Exposed individuals^a^		Odds ratio (95% confidence interval)			
	Cases	Controls	Unadjusted		Adjusted^b^	
Overall	11,502	36,472	2.04	(1.98–2.10)	1.83	(1.77–1.88)
Sex						
Men	5553	17,382	2.00	(1.92–2.08)	1.80	(1.73–1.88)
Women	5949	19,090	2.08	(2.00–2.17)	1.86	(1.78–1.94)
Age at diagnosis (years)						
< 50	257	798	2.26	(1.90–2.71)	2.20	(1.84–2.63)
50–59	1136	3493	2.31	(2.11–2.52)	2.15	(1.97–2.35)
60–69	4120	13,131	2.08	(1.99–2.18)	1.91	(1.82–2.00)
70–79	4277	13,661	1.96	(1.88–2.05)	1.72	(1.64–1.80)
≥ 80	1712	5389	1.94	(1.81–2.08)	1.68	(1.56–1.81)
Educational level^c^						
Low	4447	11,473	2.06	(1.98–2.16)	1.77	(1.69–1.86)
Middle	4941	14,800	2.12	(2.03–2.21)	1.89	(1.81–1.97)
High	1970	9814	1.93	(1.81–2.06)	1.81	(1.69–1.93)
Missing	144	385	1.90	(1.51–2.39)	1.68	(1.33–2-13)
SCC						
Overall	2966	8418	2.53	(2.39–2.69)	2.18	(2.05–2.32)
Sex						
Men	1747	4977	2.46	(2.31–2.63)	2.13	(2.00–2.28)
Women	1219	3441	2.65	(2.48–2.84)	2.26	(2.10–2.43)
Age at diagnosis (years)						
< 50	26	67	2.97	(2.46–3.59)	2.76	(2.28–3.34)
50–59	250	599	2.94	(2.65–3.26)	2.63	(2.37–2.93)
60–69	957	2696	2.64	(2.46–2.84)	2.33	(2.16–2.51)
70–79	1225	3522	2.45	(2.28–2.62)	2.07	(1.93–2.22)
≥ 80	508	1534	2.40	(2.20–2.61)	2.00	(1.84–2.19)
Educational level^c^						
Low	1246	2906	2.55	(2.38–2.73)	2.11	(1.97–2.27)
Middle	1264	3300	2.64	(2.46–2.82)	2.26	(2.11–2.43)
High	413	2118	2.42	(2.22–2.63)	2.17	(1.99–2.37)
Missing	43	94	2.34	(1.85–2.97)	2.00	(1.57–2.55)
Adenocarcinoma						
Overall	7025	23,299	1.87	(1.80–1.94)	1.70	(1.64–1.77)
Sex						
Men	3045	9980	1.80	(1.72–1.88)	1.65	(1.57–1.73)
Women	3980	13,319	1.94	(1.85–2.02)	1.75	(1.67–1.83)
Age at diagnosis (years)						
< 50	198	629	2.16	(1.81–2.59)	2.11	(1.76–2.53)
50–59	747	2455	2.14	(1.96–2.34)	2.01	(1.84–2.20)
60–69	2667	8815	1.92	(1.83–2.02)	1.78	(1.69–1.88)
70–79	2482	8342	1.78	(1.69–1.87)	1.58	(1.50–1.67)
≥ 80	931	3058	1.74	(1.62–1.88)	1.53	(1.42–1.65)
Educational level^c^						
Low	2582	7023	1.89	(1.79–1.98)	1.64	(1.56–1.73)
Middle	3024	9546	1.95	(1.86–2.05)	1.76	(1.68–1.85)
High	1341	6495	1.79	(1.67–1.91)	1.69	(1.58–1.81)
Missing	78	235	1.73	(1.37–2.18)	1.56	(1.23–1.98)
SCLC						
Overall	1982	6096	2.16	(2.01–2.32)	1.92	(1.78–2.06)
Sex						
Men	887	2746	2.11	(1.96–2.28)	1.89	(1.74–2.04)
Women	1095	3350	2.21	(2.05–2.38)	1.95	(1.80–2.10)
Age at diagnosis (years)						
< 50	46	120	2.40	(1.98–2.89)	2.30	(1.90–2.79)
50–59	223	636	2.43	(2.19–2.72)	2.25	(2.01–2.51)
60–69	727	2331	2.20	(2.03–2.39)	1.99	(1.84–2.16)
70–79	757	2322	2.07	(1.92–2.24)	1.80	(1.66–1.95)
≥ 80	229	687	2.05	(1.86–2.26)	1.76	(1.59–1.94)
Educational level^c^						
Low	818	1850	2.20	(2.03–2.38)	1.86	(1.72–2.02)
Middle	864	2521	2.25	(2.09–2.44)	1.98	(1.83–2.15)
High	277	1680	2.05	(1.87–2.25)	1.89	(1.72–2.08)
Missing	23	45	2.01	(1.59–2.56)	1.76	(1.38–2.26)

Cases and controls were matched by sex, year of birth and place of residence

Unexposed individuals are the reference group

*NSCLC* Non-small cell lung cancer, *SCC* Squamous cell carcinoma, *SCLC* Small cell lung cancer

^a^Individuals with at least one filled prescription of antibiotics recommended for the treatment of pneumonia as recorded in the Prescribed Drug Register within three years before the index date, i.e., date of lung cancer diagnosis and the corresponding date for the matched individuals free of lung cancer

^b^Adjusted for sex, year of birth, place of residence, highest attained education, previous chronic obstructive pulmonary disease diagnosis, previous use of antibiotics recommended for the treatment of pneumonia, and history of any cancer

^c^Highest attained educational level the year before the index date (i.e., date of lung cancer diagnosis and the corresponding date for the matched individuals free of lung cancer), categorized by years of formal education: ≤ 9 (low, mandatory), 10–12 (middle, upper secondary), and ≥ 13 (high, post-upper secondary)

For repeated fillings, we did not find a trend in the magnitude of the effect size by cancer stage (Table 3). However, there was an indication of a slightly pronounced magnitude of the effect size for stage III compared to stage IV disease. In separate assessments, there was no trend in the effect size by the T- and N-descriptors.

**Table 3:** Odds ratios and 95% confidence intervals for the association between a diagnosis of lung cancer and a recent history of repeated fillings (≥ 2 fillings) of antibiotics recommended for the treatment of pneumonia, by cancer stage at diagnosis, Lung Cancer DataBase Sweden, 2009–2016

		Repeated fillings (≥ 2)			
		Exposed individuals^a^		Adjusted odds ratio (95% confidence interval)^b^	
		Cases	Controls		
NSCLC, overall					
Overall		5746	14,489	2.18	(2.10–2.27)
Stage at diagnosis^c^					
	I–II	1576	3660	2.28	(2.11–2.47)
	III	1320	2996	2.54	(2.33–2.77)
	IV	2796	7692	2.02	(1.91–2.13)
	Missing	54	141	2.10	(1.39–3.18)
T-descriptor^c^					
	1	1311	3131	2.29	(2.12–2.48)
	2	1594	4169	2.03	(1.89–2.17)
	3	950	2337	2.17	(1.99–2.38)
	4	1794	4589	2.17	(2.04–2.32)
	Missing	97	263	1.87	(1.45–2.40)
N-descriptor^c^					
	0	2272	5659	2.08	(1.96–2.21)
	1	419	1156	1.93	(1.69–2.20)
	2	1659	4190	2.25	(2.10–2.40)
	3	1251	3080	2.29	(2.13–2.47)
	Missing	145	404	1.73	(1.40–2.13)
SCC					
Overall		15,877	3291	2.75	(2.53–2.99)
Stage at diagnosis^c^					
	I–II	480	962	2.53	(2.17–2.95)
	III	514	1021	3.14	(2.71–3.65)
	IV	574	1264	2.62	(2.29–3.00)
	Missing	19	44	3.41	(1.58–7.36)
T-descriptor^c^					
	1	256	473	3.03	(2.72–3.38)
	2	501	1012	2.59	(2.35–2.85)
	3	290	627	2.75	(2.46–3.06)
	4	527	1151	2.77	(2.52–3.04)
	Missing	13	28	2.46	(1.89–3.20)
N-descriptor^c^					
	0	610	1313	2.67	(2.44–2.92)
	1	145	296	2.44	(2.10–2.82)
	2	490	1007	2.86	(2.60–3.15)
	3	306	585	2.97	(2.68–3.30)
	Missing	36	90	2.20	(1.77–2.75)
Adenocarcinoma					
Overall		3419	9324	2.00	(1.90–2.10)
Stage at diagnosis^c^					
	I–II	968	2391	2.17	(1.97–2.40)
	III	598	1575	2.07	(1.83–2.33)
	IV	1826	5278	1.90	(1.78–2.04)
	Missing	27	80	1.80	(1.03–3.13)
T-descriptor^c^					
	1	920	2341	2.14	(1.97–2.32)
	2	914	2656	1.83	(1.70–1.97)
	3	517	1360	1.94	(1.76–2.13)
	4	1002	2772	1.96	(1.82–2.10)
	Missing	66	195	1.73	(1.35–2.24)
N-descriptor^c^					
	0	1444	3766	1.89	(1.77–1.98)
	1	216	710	1.73	(1.51–1.98)
	2	926	2562	2.03	(1.89–2.19)
	3	749	2054	2.11	(1.95–2.29)
	Missing	84	232	1.56	(1.26–1.93)
SCLC					
Overall		1006	2446	2.25	(2.04–2.49)
Stage at diagnosis^c^					
	I–II	47	116	1.99	(1.25–3.18)
	III	312	654	2.77	(2.29–3.35)
	IV	629	1642	2.09	(1.85–2.35)
	Missing	18	34	3.27	(1.41–7.59)
T-descriptor^c^					
	1	119	264	2.41	(2.13–2.72)
	2	170	467	2.13	(1.90–2.38)
	3	148	326	2.28	(2.02–2.58)
	4	541	1322	2.28	(2.07–2.52)
	Missing	28	67	1.96	(1.51–2.55)
N-descriptor^c^					
	0	88	306	2.12	(1.89–2.38)
	1	49	111	1.97	(1.68–2.31)
	2	399	873	2.29	(2.06–2.55)
	3	442	1080	2.34	(2.11–2.60)
	Missing	28	76	1.77	(1.41–2.21)

Cases and controls were matched by sex, year of birth and place of residence

Unexposed individuals are the reference group

*NSCLC* Non-small cell lung cancer, *SCC* Squamous cell carcinoma, *SCLC* Small cell lung cancer

^a^Individuals with repeated (≥ 2) fillings of prescriptions of antibiotics recommended for the treatment of pneumonia as recorded in the Prescribed Drug Register within three years before the index date, i.e., date of lung cancer diagnosis and the corresponding date for the matched individuals free of lung cancer

^b^Adjusted for sex, year of birth, place of residence, highest attained education, previous chronic obstructive pulmonary disease diagnosis, previous use of antibiotics recommended for the treatment of pneumonia, history of any cancer, and the other TNM-descriptors in the descriptor separate analyses (e.g., if estimating effect of exposure in subgroups of T-descriptor then the estimate is adjusted for N- and M-descriptors)

^c^Based on the tumor–node–metastasis (TNM) classification system

When assessing the exposure in the six months prior to the diagnosis only, the magnitudes of the ORs for repeated fillings became substantially pronounced compared to those unexposed and those who filled one prescription only, a pattern also present when assessing the association for different types of antibiotics from filled prescriptions (Supplementary Table S4, Supplementary Table S5). When we applied a 12-month washout period, the results remained essentially unchanged with an estimated two-fold increase in odds for a history of at least one filled prescription: NSCLC overall (OR: 1.84, 95% CI 1.77–1.90), SCC (OR: 2.11, 95% CI 1.96–2.27), adenocarcinoma (OR: 1.73, 95% CI 1.66–1.81), and SCLC (OR: 1.95, 95% CI 1.78–2.13) (Supplementary Table S6 When excluding the three months closest before the index date, the magnitudes of the ORs decreased (Supplementary Table S7). However, an indication of more pronounced ORs with an increasing number of fillings remained. Following the exclusion of individuals with a history of COPD, the estimated ORs remained virtually unchanged (Supplementary Table S8).

## Discussion

### Main findings

To the best of our knowledge, this is the first population-based study using routinely collected data that has investigated patterns of pre-diagnostic use of antibiotics as a potential early indicator of lung cancer.

Compared to individuals free of lung cancer, a higher proportion of patients with lung cancer had filled at least one prescription of antibiotics within three years prior to diagnosis, and, additionally more often had a history of repeated fillings. The odds of a recent history of at least one filled prescription was approximately two times higher among patients with lung cancer compared to individuals free of lung cancer, and the magnitude of the effect size became more pronounced with increasing numbers of filled prescriptions and with proximity to the diagnosis. Also, the magnitude of the effect size was more pronounced among younger compared to older individuals, and among patients with SCC and SCLC compared to those diagnosed with adenocarcinoma. We found no evidence in support of a trend in the magnitude of the effect size by cancer stage.

### Interpretation and comparison with other studies

Our findings of more frequent use of antibiotics prior to diagnosis in patients with lung cancer are for several reasons not surprising. First, pulmonary infections are not uncommon in the area of tumor growth and can present as the first symptom of a malignancy [3, 4]. Second, tumors and infected loci can initially be indistinguishable on radiographic evaluation [16]. Third, COPD and upper respiratory infections are more common in smokers at increased risk of lung cancer and individuals with COPD are often prescribed antibiotics for exacerbations [17, 18]. Taken together, our findings do not necessarily reflect an inappropriate prescribing of antibiotics. However, with as many as 7% of the patients with lung cancer in the present study having four or more treatment cycles (the maximum being 63) of antibiotics within three years prior to the diagnosis, our findings indicate improper clinical follow-up and reassessment for some patients, contrary to long-standing guidelines [19].

Our findings that the proportion of cases with filled antibiotic prescriptions started to increase three to four months before diagnosis as well as the pronounced effect size for being exposed in the last period prior to diagnosis indicate that early signs and symptoms of lung malignancy are commonly starting to present during this time window. Our results also corroborate the findings by *Ewing *et al. of an increasing frequency of primary care consultations approximately 80–100 days before confirmation of a diagnosis of lung cancer [20].

The reasons for the attenuation of the magnitude of the OR with increasing age are unclear, but may reflect a lower diagnostic intensity and underreporting of lung cancer among older individuals [21]. Also, the baseline occurrence of lung cancer is higher among older individuals, hence, a higher increase in absolute numbers of patients with lung cancer is required to yield the same relative change as for younger individuals. The more pronounced magnitudes of the effect sizes observed for SCC and SCLC compared to adenocarcinoma may reflect that these subtypes are generally more centrally located compared to adenocarcinomas and that they are associated with severe symptoms of pneumonia [22–24]. It may also reflect differences in smoking history: a higher proportion of patients with adenocarcinoma are never-smokers compared to those with SCC and SCLC [2, 25].

The indication of slightly attenuated magnitude of the OR for the associations between lung cancer and a history of repeated fillings for stage IV compared to stage III cancer may reflect characteristics in patients with stage III disease associated with an increased risk for infections or a presence of infection-like symptoms [26]. Stage III lung cancer is characterized by lymph node involvement or a T4-status indicating (independent of tumor size) spread to an ipsilateral lobe or invasion of heart or central parts of the lung (e.g., the carina) [27]. Patients with stage IV disease may have a small and more favorably located primary tumor without any involvement of lymph nodes, not causing infections to the same extent, and consequently, have a lower likelihood of being prescribed antibiotics before diagnosis. The descriptor specific estimates, with no trends in association for the T- and N-descriptors, bring no clarity to the observed differences between stage III and stage IV. Studies investigating the causes of diagnostic delays of lung cancer have identified factors such as inconclusive chest X-ray readings, the presence of comorbid conditions, waiting times for chest X-ray and the absence of symptoms [16, 28]. Taken together, the findings of our study do not provide evidence that a history of repeated treatment cycles of antibiotics is related to diagnostic delays, at least not as reflected in an increased likelihood of being diagnosed with more advanced disease.

Alternative explanations for our findings include a role of antibiotics as a risk factor, possibly via an influence on the immune system or host–microbiota composition [5, 6, 29]. However, for several reasons our findings are unlikely to be explained by a risk modifying effect of exposure to antibiotics. First, the latency period for the initiation and development of a malignancy is generally long. Second, the estimates did not change following the exclusion of individuals with filled prescriptions within the washout period of 12 months before the start of the observation period**.** However, we could not assess exposure before 2005, when the PDR was launched.

### Strengths and limitations

The strengths of our study included the use of data from nationwide Swedish population-based registers of high completeness and quality that minimized the risk of selection bias and misclassification bias of exposures and outcomes. The completeness and validity of prescription data in the PDR is very high, mainly because the transfer of information from community pharmacies is almost exclusively automated [13].

Several limitations need mentioning. No information was available for the indication of the prescribed antibiotics. While we restricted the analyses to antibiotics recommended for the treatment of pneumonia, it cannot be ruled out that some prescriptions were for other infections. However, because respiratory infections are one of the most common indications for antibiotic use [30], this is likely to have resulted in no or only a minor misclassification of exposure. Also, assuming a higher risk of respiratory infections in patients with lung cancer (for which the majority are current or former smokers) compared to the general population, any misclassification is likely to be more common in controls and would have biased the results toward the null. The absence of data of actual use by the patient was not a concern because the aim of this study was not to examine exposure to antibiotics as a risk factor per se. Any non-differential misclassification of the exposure or the outcome would have biased the ORs toward the null. Although most pneumonia diagnosis are made in a primary care setting, such information based on ICD coding is not available in the NPR or in the other data sources used for the purpose of the present study. However, all prescriptions made in primary care are included in the PDR, once a prescription is filled.

Because information on smoking history was available for lung cancer patients only, smoking could not be included in the adjusted models. However, information on COPD diagnosis retrieved from the NPR was included in the logistic regression models.

Our findings are likely to be generalisable to settings with similar health care systems and guidelines for the management of pneumonia and lung cancer.

## Conclusions

We found that a diagnosis of lung cancer was associated with an increased likelihood of recent pre-diagnostic fillings of antibiotic prescriptions. The likelihood became more pronounced with a greater number of fillings and with proximity to the diagnosis, further supporting the notion that infection represents an early sign of lung cancer. We found no evidence that repeated treatment cycles were associated with a diagnostic delay as reflected by cancer stage at diagnosis. Our findings further underscore the importance to rule out lung cancer following pneumonia treatment, especially in patients with a history of repeated treatment cycles.

## Supplementary Information

Below is the link to the electronic supplementary material.

Supplementary file1 (DOCX 327 KB)

## Data Availability

Data may be obtained from the registries named in the methods section and are not publicly available. The authors are, according to the law, not authorized to make the data publicly available.

## Code Availability

On request.
