# Supplementary material for: Antibiotic use prior to a lung cancer diagnosis: a population-based study
Source: Cancer Causes Control. 2021 Mar 22;32(6):597–607. doi: 10.1007/s10552-021-01413-5 (PMC8089077; doi:10.1007/s10552-021-01413-5)
Supplement: Supplementary file 1 — Supplementary file1 (DOCX 327 KB) [file 10552_2021_1413_MOESM1_ESM.docx]

**SUPPLEMENTARY MATERIAL**

| **Supplementary Table 1. Antibiotics, and corresponding anatomical therapeutic chemical codes, recommended for the treatment of pneumonia in Sweden** | |
| --- | --- |
| **Antibiotics** | **Anatomical therapeutic chemical codes** |
| Phenoxymethylpenicillin | J01CE02 |
| Amoxicillin | J01CA04 |
| Doxycycline | J01AA02 |
| Erythromycin | J01FA01 |
| Cefotaxime | J01DD01 |
| Sulfamethoxazole+trimethoprim | J01EE01 |

**
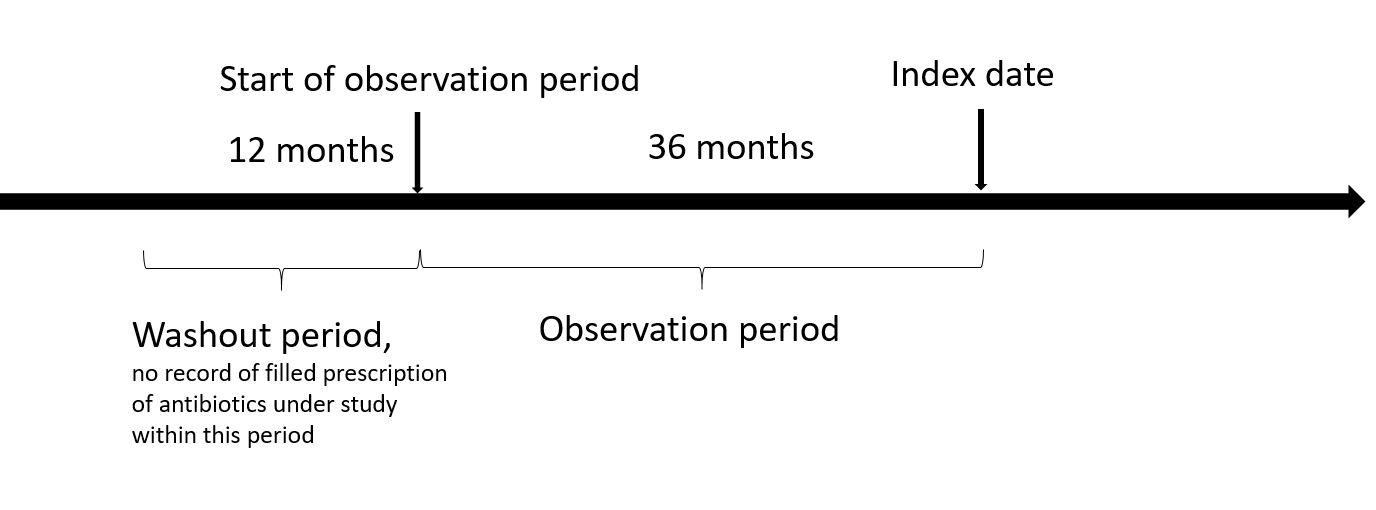
**

**Supplementary Figure 1. Study design for the sensitivity analysis when applying a washout period of 12 months**

**
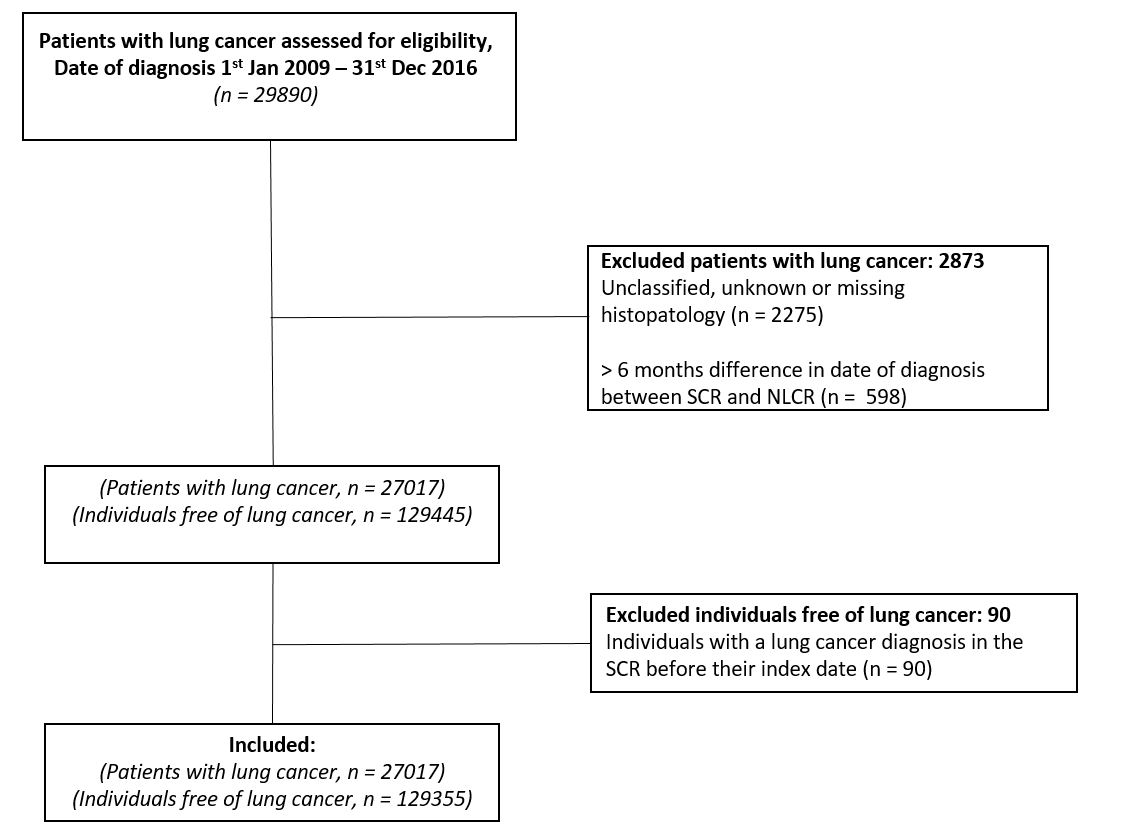
**

**Supplementary Figure 2. Flow chart for the inclusion of eligible study individuals.** Abbreviations: Swedish Cancer Register (SCR), Swedish National Lung Cancer Register (NLCR)

**
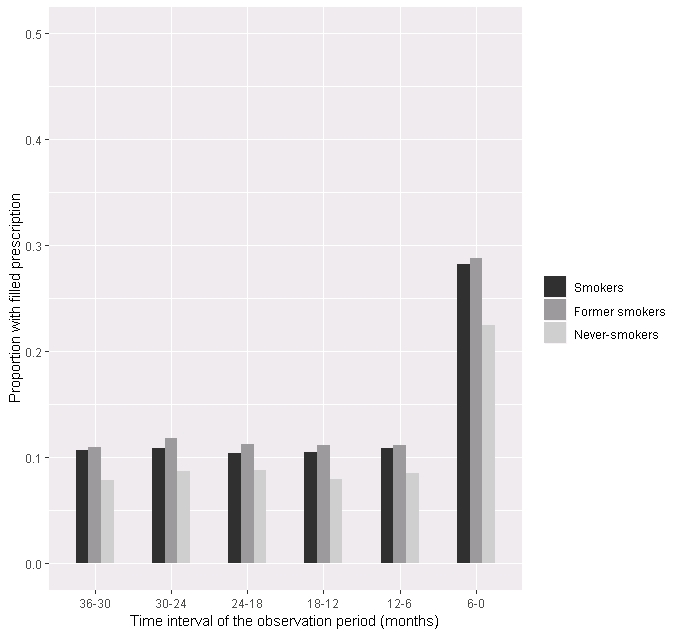
**

**Supplementary Figure 3. The proportion of cases (individuals with lung cancer) with at least one filled prescription of antibiotics recommended for the treatment of pneumonia in different time intervals of the observation period of three years prior to the diagnosis, by smoking history.** Lung Cancer DataBase Sweden, 2009-2016

**
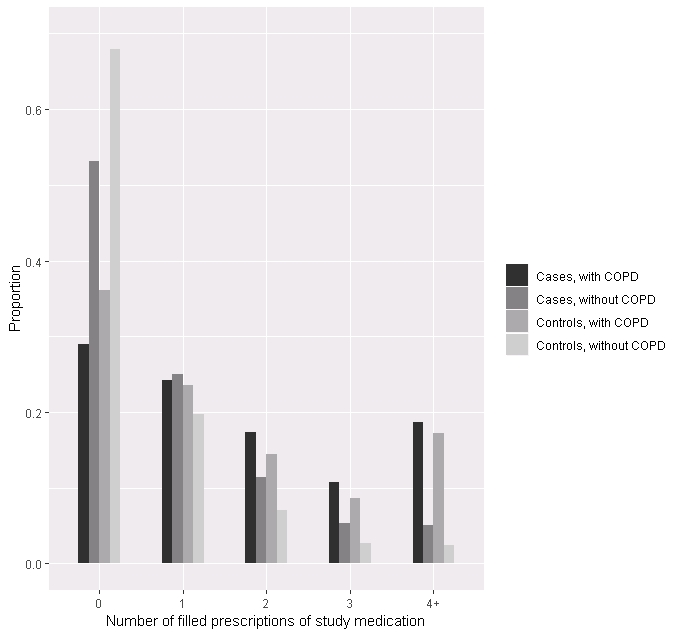
**

**Supplementary Figure 4. The proportion of cases (individuals with lung cancer) and controls (individuals free of lung cancer) by the number of recently filled prescriptions of antibiotics recommended for the treatment of pneumonia, by history of chronic obstructive pulmonary disease (COPD).** Cases and controls were matched by sex, year of birth and place of residence. Lung Cancer DataBase Sweden, 2009-2016

| **Supplementary Table 2. Odds ratios and 95% confidence intervals for the association between a diagnosis of lung cancer and at least one filled prescription of antibiotics recommended for the treatment of pneumonia in different time intervals before the diagnosis of lung cancer, Lung Cancer DataBase Sweden, 2009-2016** | | | | | | | |
| --- | --- | --- | --- | --- | --- | --- | --- |
|  |  | **Exposed individuals^a^** | | **Odds ratio (95% confidence interval)** | | | |
|  |  | **Cases** | **Controls** | **Unadjusted** | | **Adjusted^b^** | |
| **NSCLC, overall** | |  |  |  |  |  |  |
| **Time interval prior to the index date** | |  |  |  |  |  |  |
|  | <6 months | 6364 | 8668 | 4.50 | (4.34-4.67) | 4.07 | (3.92-4.23) |
|  | 6-12 months | 2500 | 8720 | 1.42 | (1.35-1.49) | 1.22 | (1.16-1.28) |
|  | 12-36 months | 7008 | 27789 | 1.30 | (1.26-1.34) | 1.16 | (1.12-1.20) |
|  |  |  |  |  |  |  |  |
| **SCC** | |  |  |  |  |  |  |
| **Time interval prior to the index date** | |  |  |  |  |  |  |
|  | <6 months | 1783 | 2060 | 5.76 | (5.34-6.22) | 4.99 | (4.61-5.40) |
|  | 6-12 months | 710 | 2034 | 1.76 | (1.61-1.93) | 1.46 | (1.33-1.61) |
|  | 12-36 months | 1774 | 6362 | 1.49 | (1.40-1.59) | 1.29 | (1.21-1.38) |
|  |  |  |  |  |  |  |  |
| **Adenocarcinoma** | |  |  |  |  |  |  |
| **Time interval prior to the index date** | |  |  |  |  |  |  |
|  | <6 months | 3724 | 5479 | 4.03 | (3.85-4.23) | 3.72 | (3..54-3.90) |
|  | 6-12 months | 1485 | 1530 | 1.32 | (1.24-1.40) | 1.16 | (1.09-1.23) |
|  | 12-36 months | 4340 | 4630 | 1.24 | (1.19-1.29) | 1.12 | (1.07-1.16) |
|  |  |  |  |  |  |  |  |
| **SCLC** | |  |  |  |  |  |  |
| **Time interval prior to the index date** | |  |  |  |  |  |  |
|  | <6 months | 1142 | 1453 | 5.05 | (4.61-5.53) | 4.49 | (4.09-4.94) |
|  | 6-12 months | 411 | 1530 | 1.32 | (1.18-1.48) | 1.14 | (1.01-1.28) |
|  | 12-36 months | 1210 | 4630 | 1.37 | (1.27-1.48) | 1.23 | (1.14-1.33) |
| ^a^ Individuals with at least one filled prescription of antibiotics recommended for the treatment of pneumonia as recorded in the Prescribed Drug Register within different time intervals of the observation period (three years [36 months] before the index date, i.e. date of lung cancer diagnosis and the corresponding date for individuals free of lung cancer).  ^b^ Adjusted sex, year of birth, place of residence, highest attained education, previous chronic obstructive pulmonary disease diagnosis, previous use of antibiotics recommended for the treatment of pneumonia, and history of any cancer.  Cases and controls were matched by sex, year of birth and place of residence.  Unexposed individuals are the reference group.  Abbreviations: Non-small cell lung cancer (NSCLC), Squamous cell carcinoma (SCC), Small cell lung cancer (SCLC). | | | | | | | |

| **Supplementary Table 3. Odds ratios and 95% confidence intervals for the association between a diagnosis of lung cancer and the number of different types of antibiotics recommended for the treatment of pneumonia from recently filled prescriptions, Lung Cancer DataBase Sweden, 2009-2016** | | | | | | | |
| --- | --- | --- | --- | --- | --- | --- | --- |
|  |  | **Exposed individuals^a^** | | **Odds ratio (95% confidence interval)** | | | |
|  |  | **Cases** | **Controls** | **Unadjusted** | | **Adjusted^b^** | |
| **NSCLC, overall** | |  |  |  |  |  |  |
| **Number of different antibiotics recommended for pneumonia^c^** |  |  |  |  |  |  |  |
|  | 0 | 0 | 0 | 1 | (ref) | 1 | (ref) |
|  | 1 | 7705 | 28320 | 1.76 | (1.70-1.82) | 1.65 | (1.60-1.71) |
|  | 2 | 3043 | 6862 | 2.88 | (2.75-3.02) | 2.43 | (2.31-2.55) |
|  | ≥3 | 754 | 1290 | 3.81 | (3.47-4.18) | 2.82 | (2.55-3.11) |
| **SCC** | |  |  |  |  |  |  |
| **Number of different antibiotics recommended for pneumonia^c^** |  |  |  |  |  |  |  |
|  | 0 | 0 | 0 | 1 | (ref) | 1 | (ref) |
|  | 1 | 1914 | 6573 | 2.09 | (1.96-2.24) | 1.92 | (1.79-2.06) |
|  | 2 | 823 | 1533 | 3.90 | (3.54-4.29) | 3.07 | (2.77-3.40) |
|  | ≥3 | 229 | 312 | 5.33 | (4.46-6.38) | 3.46 | (2.86-4.19) |
| **Adenocarcinoma** | |  |  |  |  |  |  |
| **Number of different antibiotics recommended for pneumonia^c^** |  |  |  |  |  |  |  |
|  | 0 | 0 | 0 | 1 | (ref) | 1 | (ref) |
|  | 1 | 4765 | 18053 | 1.38 | (1.22-1.55) | 1.38 | (1.22-1.56) |
|  | 2 | 1821 | 4436 | 2.56 | (2.41-2.72) | 2.21 | (2.07-2.35) |
|  | ≥3 | 439 | 810 | 3.38 | (3.00-3.82) | 2.62 | (2.31-2.98) |
| **SCLC** | |  |  |  |  |  |  |
| **Number of different antibiotics recommended for pneumonia^c^** |  |  |  |  |  |  |  |
|  | 0 | 0 | 0 | 1 | (ref) | 1 | (ref) |
|  | 1 | 1298 | 4715 | 1.83 | (1.69-1.98) | 1.70 | (1.56-1.84) |
|  | 2 | 554 | 1160 | 3.20 | (2.86-2.59) | 2.58 | (2.28-2.92) |
|  | ≥3 | 130 | 221 | 4.04 | (3.23-5.06) | 2.96 | (2.31-3.79) |
| ^a^ Individuals with fillings of at least one type of antibiotic recommended for the treatment of pneumonia as recorded in the Prescribed Drug Register within three years before the index date, i.e. date of lung cancer diagnosis and the corresponding date for individuals free of lung cancer.  ^b^ Adjusted for sex, year of birth, place of residence, highest attained education, previous chronic obstructive pulmonary disease diagnosis, previous use of antibiotics recommended for the treatment of pneumonia, and history of any cancer.  ^c^ Based on filled prescriptions of antibiotics recommended for the treatment of pneumonia as recorded in the Prescribed Drug Register within three years before the index date, i.e. date of lung cancer diagnosis and the corresponding date for individuals free of lung cancer.  Cases and controls were matched by sex, year of birth and place of residence.  Unexposed individuals are the reference group.  Abbreviations: Non-small cell lung cancer (NSCLC), Squamous cell carcinoma (SCC), Small cell lung cancer (SCLC). | | | | | | | |

| **Supplementary Table 4. Odds ratios and 95% confidence intervals for the association between a diagnosis of lung cancer and recently filled prescriptions of antibiotics recommended for the treatment of pneumonia within the six months directly before the diagnosis of lung cancer, Lung Cancer DataBase, 2009-2016** | | | | | | | |  |
| --- | --- | --- | --- | --- | --- | --- | --- | --- |
|  |  | **Exposed individuals^a^** | | **Odds ratio (95% confidence interval)** | | | | |
| **Filled prescriptions^b^** | | **Cases** | **Controls** | **Unadjusted** | | **Adjusted^c^** | | |
| **NSCLC, overall** |  |  |  |  |  |  |  | |
|  | 0 | 0 | 0 | 1 | (ref) | 1 | (ref) | |
|  | 1 | 4584 | 7131 | 3.95 | (3.79-4.12) | 3.64 | (3.49-3.80) | |
|  | ≥2 | 1780 | 1537 | 7.10 | (6.61-7.62) | 6.01 | (5.58-6.48) | |
| **SCC** |  |  |  |  |  |  |  | |
|  | 0 | 0 | 0 | 1 | (ref) | 1 | (ref) | |
|  | 1 | 1212 | 1663 | 4.86 | (4.47-5.29) | 4.38 | (4.01-4.79) | |
|  | ≥2 | 571 | 397 | 9.62 | (8.39-11.04) | 7.36 | (6.37-8.50) | |
| **Adenocarcinoma** |  |  |  |  |  |  |  | |
|  | 0 | 0 | 0 | 1 | (ref) | 1 | (ref) | |
|  | 1 | 2740 | 984 | 3.58 | (3.39-3.77) | 3.34 | (3.17-3.53) | |
|  | ≥2 | 4544 | 935 | 6.25 | (5.69-6.86) | 5.51 | (5.00-6.08) | |
| **SCLC** |  |  |  |  |  |  |  | |
|  | 0 | 0 | 0 | 1 | (ref) | 1 | (ref) | |
|  | 1 | 825 | 1193 | 4.45 | (4.02-4.92) | 4.11 | (3.69-4.58) | |
|  | ≥2 | 317 | 260 | 7.75 | (6.52-9.22) | 6.61 | (5.48-7.96) | |
| ^a^ Individuals with one filled prescription, or repeated fillings (≥2), of antibiotics recommended for the treatment of pneumonia as recorded in the Prescribed Drug Register within the six months before the index date, i.e. date of lung cancer diagnosis and the corresponding date for individuals free of lung cancer.  ^b^ Based on filled prescriptions of antibiotics recommended for the treatment of pneumonia as recorded in the Prescribed Drug Register within the six months before the index date, i.e. date of lung cancer diagnosis and the corresponding date for individuals free of lung cancer.  ^c^ Adjusted for sex, year of birth, place of residence, highest attained education, previous chronic obstructive pulmonary disease diagnosis, previous use of antibiotics recommended for the treatment of pneumonia, and history of any cancer.  Cases and controls were matched by sex, year of birth and place of residence.  Unexposed individuals are the reference group.  Abbreviations: Non-small cell lung cancer (NSCLC), Squamous cell carcinoma (SCC), Small cell lung cancer (SCLC). | | | | | | | | |

| **Supplementary Table 5. Odds ratios and 95% confidence intervals for the association between a diagnosis of lung cancer and the number of different types of antibiotics recommended for the treatment of pneumonia from recently filled prescriptions within six months before the diagnosis of lung cancer, Lung Cancer DataBase Sweden, 2009-2016** | | | | | | | |  |
| --- | --- | --- | --- | --- | --- | --- | --- | --- |
|  |  | **Exposed individuals^a^** | | **Odds ratio (95% confidence interval)** | | | | |
|  |  | **Cases** | **Controls** | **Unadjusted** | | **Adjusted^b^** | | |
| **NSCLC** | |  |  |  |  |  |  | |
| **Number of different antibiotics for pneumonia^c^** | |  |  |  |  |  |  | |
|  | 0 | 0 | 0 | 1 | (ref) | 1 | (ref) | |
|  | 1 | 5190 | 8018 | 3.97 | (3.82-4.13) | 3.62 | (3.47-3.77) | |
|  | ≥2 | 1174 | 650 | 11.19 | (10.13-12.36) | 9.67 | (8.72-10.72) | |
|  |  |  |  |  |  |  |  | |
| **SCC** | |  |  |  |  |  |  | |
| **Number of different antibiotics for pneumonia^c^** | |  |  |  |  |  |  | |
|  | 0 | 0 | 0 | 1 | (ref) | 1 | (ref) | |
|  | 1 | 1419 | 1879 | 5.03 | (4.64-5.45) | 4.43 | (4.08-4.82) | |
|  | ≥2 | 364 | 181 | 13.75 | (11.38-16.61) | 10.55 | (8.66-12.85) | |
|  |  |  |  |  |  |  |  | |
| **Adenocarcinoma** | |  |  |  |  |  |  | |
| **Number of different antibiotics for pneumonia^c^** | |  |  |  |  |  |  | |
|  | 0 | 0 | 0 | 1 | (ref) | 1 | (ref) | |
|  | 1 | 3066 | 5092 | 3.57 | (3.40-3.75) | 3.30 | (3.14-3.48) | |
|  | ≥2 | 658 | 387 | 10.19 | (8.95-11.60) | 9.27 | (8.10-10.61) | |
|  |  |  |  |  |  |  |  | |
| **SCLC** | |  |  |  |  |  |  | |
| **Number of different antibiotics for pneumonia^c^** | |  |  |  |  |  |  | |
|  | 0 | 0 | 0 | 1 | (ref) | 1 | (ref) | |
|  | 1 | 928 | 1329 | 4.49 | (4.07-4.94) | 4.09 | (3.69-4.54) | |
|  | ≥2 | 214 | 124 | 11.10 | (8.82-13.98) | 9.65 | (7.55-12.34) | |
| ^a^ Individuals with fillings of one or different (≥2) types of antibiotics recommended for the treatment of pneumonia as recorded in the Prescribed Drug Register within the six months before the index date, i.e. date of lung cancer diagnosis and the corresponding date for individuals free of lung cancer.  ^b^ Based on filled prescriptions of antibiotics recommended for the treatment of pneumonia as recorded in the Prescribed Drug Register within the six months before the index date, i.e. date of lung cancer diagnosis and the corresponding date for individuals free of lung cancer.  ^c^ Adjusted for sex, year of birth, place of residence, highest attained education, previous chronic obstructive pulmonary disease diagnosis, previous use of antibiotics recommended for the treatment of pneumonia, and history of any cancer.  Cases and controls were matched by sex, year of birth and place of residence.  Unexposed individuals are the reference group.  Abbreviations: Non-small cell lung cancer (NSCLC), Squamous cell carcinoma (SCC), Small cell lung cancer (SCLC). | | | | | | | | |

| **Supplementary Table 6. Odds ratios and 95% confidence intervals for the association between a diagnosis of lung cancer and a recent history of at least one filled prescription of antibiotics recommended for the treatment of pneumonia, Lung Cancer DataBase Sweden, 2009-2016. Twelve months incident patients only, i.e. individuals with a history of a filled prescription in the 12 months period prior to the start of the observation period (three years before the date of lung cancer diagnosis and the corresponding date for individuals free of lung cancer) were excluded from the study population** | | | | | | | |  |
| --- | --- | --- | --- | --- | --- | --- | --- | --- |
|  |  | **Exposed individuals^a^** | | **Odds ratio (95% confidence interval)** | | | | |
|  |  | **Cases** | **Controls** | **Unadjusted** | | **Adjusted^b^** | | |
| **NSCLC, overall** | | 7862 | 25471 | 1.99 | (1.92-2.06) | 1.84 | (1.77-1.90) | |
| Adenocarcinoma | | 4850 | 16378 | 1.85 | (1.77-1.93) | 1.73 | (1.66-1.81) | |
| SCC | | 2000 | 5853 | 2.38 | (2.22-2.55) | 2.11 | (1.96-2.27) | |
|  | Men | 3941 | 12374 | 1.97 | (1.88-2.07) | 1.85 | (1.76-1.94) | |
|  | Women | 3921 | 13097 | 2.00 | (1.88-2.07) | 1.83 | (1.74-1.93) | |
| **SCLC** | | 1338 | 4276 | 2.11 | (1.94-2.29) | 1.95 | (1.78-2.13) | |
|  | Men | 630 | 1929 | 2.15 | (1.91-2.43) | 2.06 | (1.82-2.34) | |
|  | Women | 708 | 2347 | 2.07 | (1.84-2.33) | 1.84 | (1.62-2.08) | |
| ^a^ Individuals with at least one filled prescription of antibiotics recommended for the treatment of pneumonia as recorded in the Prescribed Drug Register within three years before the index date, i.e. date of lung cancer diagnosis and the corresponding date for individuals free of lung cancer.  ^b^ Adjusted for sex, year of birth, place of residence, highest attained education, previous chronic obstructive pulmonary disease diagnosis, previous use of antibiotics recommended for the treatment of pneumonia, and history of any cancer.  Cases and controls were matched by sex, year of birth and place of residence.  Unexposed individuals are the reference group.  Abbreviations: Non-small cell lung cancer (NSCLC), Squamous cell carcinoma (SCC), Small cell lung cancer (SCLC). | | | | | | | | |

| **Supplementary Table 7. Odds ratios and 95% confidence intervals for the association between a diagnosis of lung cancer and the number of recently filled prescriptions of antibiotics recommended for the treatment of pneumonia, Lung Cancer DataBase Sweden, 2009-2016. Exposure in the three months before the diagnosis of lung cancer was not considered** | | | | | | | |  |
| --- | --- | --- | --- | --- | --- | --- | --- | --- |
|  |  | **Exposed Individuals^a^** | | **Odds ratio (95% confidence interval)** | | | | |
|  |  | **Cases** | **Controls** | **Unadjusted** | | **Adjusted^b^** | | |
| **NSCLC** | |  |  |  |  |  |  | |
| **≥1 filled prescription with antibiotics recommended for pneumonia^c^** | | 8885 | 34457 | 1.39 | (1.35-1.43) | 1.20 | (1.16-1.24) | |
| **Number of filled prescriptions of antibiotics**  **recommended for pneumonia^c^** | |  |  |  |  |  |  | |
|  | 0 | 0 | 0 | 1 | (ref) | 1 | (ref) | |
|  | 1 | 4794 | 21169 | 1.22 | (1.18-1.26) | 1.13 | (1.08-1.17) | |
|  | 2 | 2094 | 7514 | 1.50 | (1.42-1.58) | 1.30 | (1.24-1.38) | |
|  | 3 | 929 | 2941 | 1.71 | (1.59-1.85) | 1.36 | (1.26-1.48) | |
|  | ≥4 | 1068 | 2833 | 2.02 | (1.88-2.18) | 1.34 | (1.24-1.46) | |
|  |  |  |  |  |  |  |  | |
| **SCC** | |  |  |  |  |  |  | |
| **≥1 filled prescription with antibiotics recommended for pneumonia^c^** | | 2271 | 7938 | 1.64 | (1.54-1.74) | 1.36 | (1.27-1.45) | |
| **Number of filled prescriptions of antibiotics**  **recommended for pneumonia^c^** | |  |  |  |  |  |  | |
|  | 0 | 0 | 0 | 1 | (ref) | 1 | (ref) | |
|  | 1 | 1137 | 4962 | 1.31 | (1.22-1.42) | 1.19 | (1.10-1.29) | |
|  | 2 | 541 | 1648 | 1.88 | (1.69-2.09) | 1.54 | (1.38-1.73) | |
|  | 3 | 265 | 650 | 2.35 | (2.02-2.72) | 1.83 | (1.56-2.14) | |
|  | ≥4 | 328 | 678 | 2.76 | (2.41-3.17) | 1.63 | (1.40-1.89) | |
|  |  |  |  |  |  |  |  | |
| **Adenocarcinoma** | |  |  |  |  |  |  | |
| **≥1 filled prescription with antibiotics recommended for pneumonia^c^** | | 5494 | 22038 | 1.32 | (1.27-1.37) | 1.15 | (1.11-1.20) | |
| **Number of filled prescriptions of antibiotics**  **recommended for pneumonia^c^** | |  |  |  |  |  |  | |
|  | 0 | 0 | 0 | 1 | (ref) | 1 | (ref) | |
|  | 1 | 3034 | 13451 | 1.19 | (1.14-1.25) | 1.10 | (1.05-1.16) | |
|  | 2 | 1295 | 4887 | 1.40 | (1.31-1.49) | 1.24 | (1.16-1.33) | |
|  | 3 | 560 | 1925 | 1.55 | (1.40-1.71) | 1.25 | (1.13-1.38) | |
|  | ≥4 | 605 | 1775 | 1.79 | (1.63-1.97) | 1.23 | (1.11-1.37) | |
|  |  |  |  |  |  |  |  | |
| **SCLC** | |  |  |  |  |  |  | |
| **≥1 filled prescription with antibiotics recommended for pneumonia^c^** | | 1512 | 5789 | 1.42 | (1.32-1.52) | 1.21 | (1.12-1.31) | |
| **Number of filled prescriptions of antibiotics**  **recommended for pneumonia^c^** | |  |  |  |  |  |  | |
|  | 0 | 0 | 0 | 1 | (ref) | 1 | (ref) | |
|  | 1 | 829 | 3555 | 1.27 | (1.16-1.38) | 1.14 | (1.04-1.26) | |
|  | 2 | 329 | 1286 | 1.38 | (1.21-1.57) | 1.16 | (1.01-1.33) | |
|  | 3 | 157 | 487 | 1.75 | (1.45-2.11) | 1.31 | (1.07-1.61) | |
|  | ≥4 | 197 | 461 | 2.34 | (1.97-2.79) | 1.50 | (1.23-1.83) | |
| ^a^ Individuals with at least one filled prescription of antibiotics recommended for the treatment of pneumonia as recorded in the Prescribed Drug Register within 4-36 months before the index date, i.e. date of lung cancer diagnosis and the corresponding date for individuals free of lung cancer.  ^b^ Adjusted for sex, year of birth, place of residence, highest attained education, previous chronic obstructive pulmonary disease diagnosis, previous use of antibiotics recommended for the treatment of pneumonia, and history of any cancer.  ^c^ Based on filled prescriptions of antibiotics recommended for the treatment of pneumonia as recorded in the Prescribed Drug Register within 4-36 months before the index date, i.e. date of lung cancer diagnosis and the corresponding date for individuals free of lung cancer.  Cases and controls were matched by sex, year of birth and place of residence.  Unexposed individuals are the reference group.  Abbreviations: Non-small cell lung cancer (NSCLC), Squamous cell carcinoma (SCC), Small cell lung cancer (SCLC). | | | | | | | | |

| **Supplementary Table 8. Odds ratios and 95% confidence intervals for the association between a diagnosis of lung cancer and the number of recently filled prescriptions of antibiotics recommended for the treatment of pneumonia, Lung Cancer DataBase Sweden, 2009-2016. Individuals with a history of chronic obstructive pulmonary disease were excluded from the study population** | | | | | | | |  |
| --- | --- | --- | --- | --- | --- | --- | --- | --- |
|  |  | **Exposed individuals^a^** | | **Odds ratio (95% confidence interval)** | | | | |
|  |  | **Cases** | **Controls** | **Unadjusted** | | **Adjusted^b^** | | |
| **NSCLC** | |  |  |  |  |  |  | |
| **≥1 filled prescription with antibiotics recommended for pneumonia^c^** | | 9526 | 30453 | 1.89 | (1.83-1.95) | 1.87 | (1.81-1.93) | |
| **Number of filled prescriptions of antibiotics**  **recommended for pneumonia^c^** | |  |  |  |  |  |  | |
|  | 0 | 0 | 0 | 1 | (ref) | 1 | (ref) | |
|  | 1 | 5087 | 18750 | 1.64 | (1.58-1.70) | 1.63 | (1.57-1.70) | |
|  | 2 | 2314 | 6643 | 2.10 | (1.99-2.21) | 2.09 | (1.98-2.20) | |
|  | 3 | 1092 | 2631 | 2.54 | (2.36-2.73) | 2.51 | (2.33-2.71) | |
|  | ≥4 | 1033 | 2429 | 2.58 | (2.39-2.78) | 2.56 | (2.36-2.76) | |
| **SCC** | |  |  |  |  |  |  | |
| **≥1 filled prescription with antibiotics recommended for pneumonia^c^** | | 2222 | 6423 | 2.25 | (2.10-2.40) | 2.20 | (2.07-2.38) | |
| **Number of filled prescriptions of antibiotics**  **recommended for pneumonia^c^** | |  |  |  |  |  |  | |
|  | 0 | 0 | 0 | 1 | (ref) | 1 | (ref) | |
|  | 1 | 1146 | 4015 | 1.86 | (1.71-2.01) | 1.85 | (1.71-2.01) | |
|  | 2 | 518 | 1355 | 2.47 | (2.21-2.76) | 2.46 | (2.20-2.75) | |
|  | 3 | 282 | 534 | 3.51 | (3.01-4.09) | 3.49 | (2.98-4.08) | |
|  | ≥4 | 276 | 519 | 3.49 | (2.99-4.07) | 3.41 | (2.91-3.99) | |
| **Adenocarcinoma** | |  |  |  |  |  |  | |
| **≥1 filled prescription with antibiotics recommended for pneumonia^c^** | | 6030 | 19998 | 1.77 | (1.71-1.84) | 1.76 | (1.69-1.83) | |
| **Number of filled prescriptions of antibiotics**  **recommended for pneumonia^c^** | |  |  |  |  |  |  | |
|  | 0 | 0 | 0 | 1 | (ref) | 1 | (ref) | |
|  | 1 | 3251 | 12243 | 1.56 | (1.49-1.64) | 1.56 | (1.49-1.63) | |
|  | 2 | 1463 | 4415 | 1.94 | (1.82-2.08) | 1.93 | (1.81-2.07) | |
|  | 3 | 381 | 1757 | 2.30 | (2.10-2.53) | 2.29 | (2.08-2.52) | |
|  | ≥4 | 635 | 1583 | 2.37 | (2.15-.61) | 2.38 | (2.16-2.63) | |
| **SCLC** | |  |  |  |  |  |  | |
| **≥1 filled prescription with antibiotics recommended for pneumonia^c^** | | 1556 | 4894 | 1.95 | (1.80-2.11) | 1.95 | (1.80-2.11) | |
| **Number of filled prescriptions of antibiotics**  **recommended for pneumonia^c^** | |  |  |  |  |  |  | |
|  | 0 | 0 | 0 | 1 | (ref) | 1 | (ref) | |
|  | 1 | 828 | 2987 | 1.70 | (1.55-1.87) | 1.73 | (1.55-1.90) | |
|  | 2 | 387 | 1104 | 2.16 | (1.90-2.45) | 2.18 | (1.91-2.48) | |
|  | 3 | 167 | 426 | 2.42 | (2.01-2.93) | 2.48 | (2.05-3.01) | |
|  | ≥4 | 174 | 377 | 2.83 | (2.34-3.42) | 2.97 | (2.44-3.61) | |
| ^a^ Individuals with at least one filled prescription of antibiotics recommended for the treatment of pneumonia as recorded in the Prescribed Drug Register within three years before the index date, i.e. date of lung cancer diagnosis and the corresponding date for individuals free of lung cancer.  ^b^ Adjusted for sex, year of birth, place of residence, highest attained education, previous use of antibiotics recommended for the treatment of pneumonia, and history of any cancer.  ^c^ Based on filled prescriptions of antibiotics recommended for the treatment of pneumonia as recorded in the Prescribed Drug Register within three years before the index date, i.e. date of lung cancer diagnosis and the corresponding date for individuals free of lung cancer.  Cases and controls were matched by sex, year of birth and place of residence.  Unexposed individuals are the reference group.  Abbreviations: Non-small cell lung cancer (NSCLC), Squamous cell carcinoma (SCC), Small cell lung cancer (SCLC). | | | | | | | | |
